# Anthelmintic Baiting of Foxes against *Echinococcus multilocularis* in Small Public Area, Japan

**DOI:** 10.3201/eid2808.212016

**Published:** 2022-08

**Authors:** Kohji Uraguchi, Takao Irie, Hirokazu Kouguchi, Azusa Inamori, Mariko Sashika, Michito Shimozuru, Toshio Tsubota, Kinpei Yagi

**Affiliations:** Hokkaido Institute of Public Health, Sapporo, Japan (K. Uraguchi, T. Irie, H. Kouguchi, K. Yagi);; Hokkaido University, Sapporo (A. Inamori, M. Sashika, M. Shimozuru, T. Tsubota)

**Keywords:** *Echinococcus multilocularis*, anthelmintic baiting, red fox, Japan, zoonoses, vector-borne infections, parasites

## Abstract

We distributed anthelmintic baits on a university campus in Japan inhabited by foxes infected with *Echinococcus multilocularis* to design an effective baiting protocol for small public areas. High-density baiting can reduce the risk for human exposure to the parasite to near zero. However, monthly baiting is recommended to maintain this effect.

Alveolar echinococcosis is a potentially fatal disease caused by the larvae of the *Echinococcus multilocularis* tapeworm, which is widely distributed in the Northern Hemisphere (*1*). This parasite primarily depends on red foxes as definitive hosts, along with small mammals (mainly *Myodes rufocanus* gray-backed voles) as intermediate hosts in Japan (*2*). Human infection occurs by accidental ingestion of the parasite eggs excreted through the feces of definitive hosts.

Field trials aimed at reducing the rate of *E. multilocularis* infection in foxes through the distribution of praziquantel-containing baits have been conducted in Europe and Japan (*3*–*7*). These studies showed that anthelmintic baiting over a large area effectively reduces the infection rate in foxes; however, in most cases, eradicating the parasite from the area is difficult. Urban fox populations have increased in many countries in recent decades. In Hokkaido, Japan, foxes invade and breed on smaller spatial scales, such as university campuses and zoos in urban areas (*8*). Several deaths in zoo animals infected with echinococcosis have also been reported (*9*). Reducing the risk for infection among workers, students or visitors, and zoo animals has become an important issue for facility managers. Anthelmintic baiting may be an efficient measure against echinococcosis in such areas with many users on a small spatial scale. However, the effect of baiting on such small public areas has not been widely examined (*10*).

We conducted this study to provide a basic dataset for designing an effective baiting protocol for small public areas. We investigated the effect of high-density baiting on contamination by *E. multilocularis* eggs on a university campus in Japan.

## The Study

**Figure 1:**
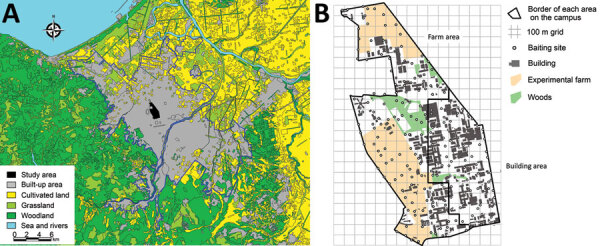
Study area for anthelmintic baiting experiment to control *Echinococcus multilocularis* tapeworms in Sapporo, Japan. A) Land use map around the study area, the Hokkaido University campus. The bold blue line shows the border of the urban area of Sapporo. B) Baiting sites and locations of buildings, farm areas, and wooded areas on the Hokkaido University campus.

The study was conducted on the Hokkaido University campus (an area of 1.8 km^2^) in an urban area of Sapporo, Japan (Figure 1, panel A). We evenly distributed anthelmintic baits manually by using 100-m grids on a map (Figure 1, panel B). We structured bait distribution into 2 phases. In phase 1 (August 2014–early July 2016), we distributed 100 baits/km^2^ monthly across the campus during the summer and fall of 2014 and 2015. In phase 2 (late July 2016–December 2018), we distributed baits monthly throughout the year. We excluded the building area (Figure 1, panel B) from baiting in this phase because the bait consumption and frequency of foxes in the camera survey in this area were relatively low compared with the farm area in phase 1. We reduced the baiting area to ≈70% of the campus (an area of 1.3 km^2^), and the density of baits on the campus decreased to ≈70/km^2^. These baiting densities in this study are higher than those used in previous studies. We prepared anthelmintic baits for this study by mixing praziquantel with fishmeal and 2 types of edible fats, which we formed into pellets containing 50 mg praziquantel each (*11*).

**Figure 2:**
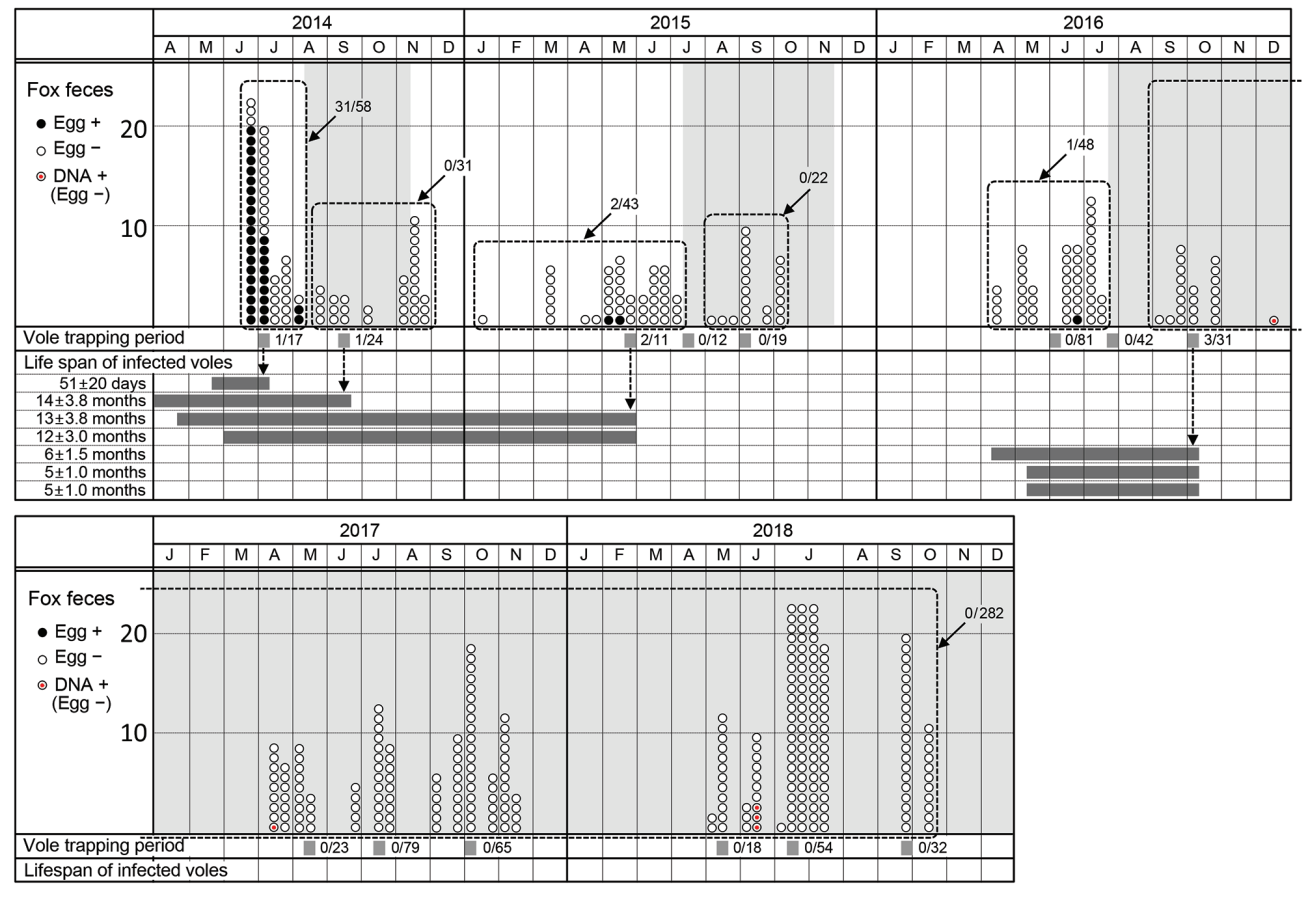
*Echinococcus multilocularis* tapeworm prevalence in foxes and voles at Hokkaido University campus, Sapporo, Japan, June 2014–October 2018. In the fox feces section, circles in each month show the fecal samples collected at the beginning, middle, and end of the month (88 fecal samples were collected in the middle of July 2018). Black circles indicate fecal samples that were *E. multilocularis* egg–positive. White circles indicate fecal samples that were *E. multilocularis* egg–negative. Circled red dots show fecal samples that were egg-negative and positive for *E. multilocularis*–specific copro-DNA. Fractions indicate the egg-positive rate of fecal samples collected during each period enclosed by a dashed line. Light gray shaded areas indicate the baiting periods. In the vole trapping period section, gray strips show the vole trapping periods. Fractions indicate the infection rate of *E. multilocularis* in *Myodes rufocanus* voles in each trapping period. In the life span of infected voles section, dark gray bars show the life span of 7 infected *M. rufocanus* voles, estimated from the age on the day of trapping (+ SD of the z score). +, positive; –, negative.

To determine the effect of baiting, we detected *E. multilocularis* eggs in fox feces, we collected fox fecal samples on campus mainly during the snowless season (Figure 2). We examined parasite eggs in all fecal samples by using a sugar flotation method with 1 g of feces (*12*), and then we molecularly analyzed the species of detected taeniid egg by using PCR/sequencing of the cytochrome *c* oxidase subunit 1 and nuclear U1 spliceosomal RNA genes (*13*). We also examined fecal samples collected in phase 2 for copro-DNA derived from the body of an adult *E. multilocularis* worm by using 3 g of feces (*14*). We recorded the number of fox fecal samples collected on the campus and presence or absence of *E. multilocularis* eggs or DNA in the samples (Figure 2). Before the first bait campaign, 53.4% (31/58) of the collected feces contained eggs. In phase 1, we collected 144 fecal samples, 2.1% (3/144) of which were egg-positive. We identified all detected eggs as *E. multilocularis* by analyzing the cytochrome *c* oxidase subunit 1 and nuclear U1 spliceosomal RNA gene sequences. We found no egg-positive feces during the baiting period (Figure 2). In phase 2, none of the 282 fecal samples collected during September 2016–October 2018 contained eggs (Table). However, we detected *E. multilocularis*–specific DNA by using the copro-DNA test on 5 fecal samples collected in phase 2 (Figure 2).

**Table:** *Echinococcus multilocularis* egg–positive rate of fox fecal samples collected on Hokkaido University campus, Sapporo, Japan, June 2014–October 2018

Baiting phase	Fecal sample collection period	Implementation of baiting	Total no. fecal samples	No. egg-positive fecal samples	Egg-positive rate of fecal samples, %
Pre-survey	2014 Jun–Aug	Before bating	58	31	53.4
Phase 1	2014 Aug–Nov	Baiting	31	0	0.0
	2015 Jan–Jul	Nonbaiting	43	2	4.7
	2015 Aug–Oct	Baiting	22	0	0.0
	2016 Apr–Jul	Nonbaiting	48	1	2.1
Phase 2	2016 Sep–2018 Oct	Baiting	282	0	0.0

We investigated the prevalence of *E. multilocularis* larvae in intermediate hosts on the campus. We set 150–250 traps (H.B. Sherman Traps Inc., https://www.shermantraps.com) for 3 consecutive days in the spring, summer, and fall seasons of 2014–2018, except for the spring of 2014 (Figure 2). We dissected all captured mammals and examined them macroscopically for lesions in the liver and other organs. We investigated lesions for *E. multilocularis* metacestode tissues by examining morphologic features. We determined the age of *M. rufocanus* voles, the most important intermediate host in Hokkaido (*2*), by examining the shape and root ratio of the molars (*15*). In total, we captured 649 small mammals of 6 species on the campus (Appendix Table). Seven of the 508 *M. rufocanus* voles were infected with *E. multilocularis*. The age of all *M. rufocanus* voles ranged from 20 days to 16 months. Of these, 6.8% were older than 12 months. The ages (+ SD of the z score) of the 7 infected voles were 51 + 20 days and 5 + 1.0 (2 individual voles), 6 + 1.5, 12 + 3.0, 13 + 3.8, and 14 + 3.8 months. Judging from their ages, we determined that the lifespan of all infected voles included the nonbaiting period (Figure 2). None of the 286 voles born in phase 2 were infected. These results show that if egg-positive fox feces are present during the nonbaiting period, voles can be infected with *E. multilocularis* worms and remain a source for infection of foxes for a year or more.

## Conclusions

Although the egg-positive rate of fecal samples is not equivalent to the infection rate in foxes, this rate directly represents the risk for exposure to the parasite eggs when university staff and students come into contact with the feces on campus. The goal of baiting on the campus is not to reduce the infection rate in foxes, but to reduce the egg-positive rate to near zero to prevent human infection with *E. multilocularis* tapeworms within the campus.

In this study, high-density, monthly baiting nearly eradicated the parasite eggs in a campus for >2 years. The effectiveness of high-density baiting has also been demonstrated in Europe, although the evaluation methods were different (*3*,*10*). In contrast, when the baiting was suspended, egg-positive feces were found again in 6–7 months, possibly because of the long lifespan of the intermediate host. Even after monthly baiting for 22 months, longer than the generation time of voles, DNA-positive feces were found in June 2018, possibly because of migrating foxes. These results suggest that preventing reinfection of foxes is difficult, even in a small area. However, even if reinfection occurs, monthly baiting will probably eliminate the parasites before the foxes excrete the parasite eggs, because the monthly interval is approximately the same as the prepatent period for *E. multilocularis* tapeworms. Eradicating *E. multilocularis* tapeworms from an area is difficult, but eradicating the parasite eggs may be possible.

These findings are subject to limitations because results may not be completely generalizable. Further studies are needed to identify individual feces using genetic analysis to achieve a more detailed understanding of the mechanism of small area baiting. In summary, high-density, monthly baiting is effective for preventing human infection with *E. multilocularis* tapeworms within small public areas.

Appendix Additional information about anthelmintic baiting of foxes against *Echinococcus multilocularis* in small public area, Japan.
