# Supplementary material for: Anthelmintic Baiting of Foxes against Echinococcus multilocularis in Small Public Area, Japan
Source: Emerg Infect Dis. 2022 Aug;28(8):1677–80. doi: 10.3201/eid2808.212016 (PMC9328911; doi:10.3201/eid2808.212016)
Supplement: Appendix — Additional information about anthelmintic baiting of foxes against Echinococcus multilocularis in small public area, Japan. [file 21-2016-Techapp-s1.pdf]

Article DOI: https://doi.org/10.3201/eid2808.212016

Anthelmintic Baiting of Foxes against *Echinococcus multilocularis* in Small Public Area, Japan

Appendix

Appendix Table. Prevalence of *Echinococcus multilocularis* in small mammals on Hokkaido University campus*

	2014 (450 TN)		2015 (450 TN)			2016 (750 TN)			2017 (750 TN)			2018 (750 TN)			
Species	Jul	Sep	May	Jul	Sep	May	Jul	Oct	May	Jul	Oct	May	Jul	Oct	Total
Myodes rufocanus	1/17	1/24	2/11	0/12	0/19	0/81	0/42	3/31	0/23	0/79	0/65	0/18	0/54	0/32	7/508
Apodemus speciosus	0/31	0/11	0/15	0/3	0/7	0/6	0/1	0/2	–	–	0/3	–	0/7	–	0/86
Apodemus argenteus	–	–	–	–	–	–	–	–	–	–	–	0/2	–	–	0/2
Rattus norvegicus	0/1	0/7	–	–	0/2	–	0/3	0/22	0/1	–	0/1	–	–	0/5	0/42
Sorex unguiculatus	–	0/1	–	–	–	–	–	0/1	0/1	0/1	0/6	–	–	–	0/10
Sorex cecutiens	–	–	–	–	–	–	0/1	–	–	–	–	–	–	–	0/1

*TN, trap night.
